# Supplementary material for: Spatiotemporal Distribution of Dengue and Chikungunya in the Hindu Kush Himalayan Region: A Systematic Review
Source: Int J Environ Res Public Health. 2020 Sep 12;17(18):6656. doi: 10.3390/ijerph17186656 (PMC7560004; doi:10.3390/ijerph17186656)
Supplement: Supplementary file 1 [file ijerph-17-06656-s001.zip › Supplement S15_Search terms.docx]

**Spatio-temporal distribution of Dengue and Chikungunya in Hindu Kush Himalayan region: A systematic review**

**Detail list of search terms**

**TOPIC**

Chikungunya OR dengue OR “breakbone fever*” OR “break-bone fever*” OR *aedes*

**AND**

himalaya* OR “hindu kush” OR Hindukush* OR nepal* OR bhutan* OR ganges OR “amu darya” OR amudarja OR Brahmaputra OR indus OR Irrawaddy OR Ayeyarwad*y OR irawadi OR Mekong OR Salween OR tarim OR Yangtze OR “yellow river” OR “huang he” OR “Chittagong Hill*” OR Tibet* OR Diqing OR Nujiang OR “Dali prefecture*” OR ganzi OR garze OR aba OR ngawa OR Liangshan OR gannan OR wuwei OR zhangye OR kashigar OR kaxgar OR kashgar OR kezilesu OR kizilsu OR hetian OR hotan OR altai OR altay OR Qinghai OR “arunachal Pradesh” OR “Himachal Pradesh” OR Kashmir OR uttarakhand OR assam OR asam OR Manipur OR maghalaya OR Mizoram OR Nagaland OR Sikkim OR Tripura OR darjeeling OR Kachin OR chin OR shan OR rakkhain OR Rakhine OR thanlwin OR Karakoram OR “Hindu raj” OR “Qogir” OR “safed koh” OR “spin ghar” OR kirthar OR “salt range” OR Suleiman OR jammu OR ((“north western frontier” OR “northwest frontier” OR “federally administered tribal” OR “northern areas” OR Bal?chistan OR Baluchi) AND Pakistan) OR Badakhshan OR Badghis OR Baghlan OR Balkh OR Bamyan OR Daykundi OR Faryab OR Ghazni OR Ghor OR Jowzjan OR Kabul OR Kapisa OR Khost OR Kunar OR Kunduz OR Laghman OR Logar OR Maidan Wardak OR Nangarhar OR Nuristan OR Paktia OR Paktika OR Panjshir OR Parwan OR Samangan OR Sar-e Pol OR Takhar OR Urozgan OR Zabul

**Not**

*delta
